# TenseMusic: An automatic prediction model for musical tension

**DOI:** 10.1371/journal.pone.0296385

**Published:** 2024-01-19

**Authors:** Alice Vivien Barchet, Johanna M. Rimmele, Claire Pelofi

**Affiliations:** 1 Department of Cognitive Neuropsychology, Max Planck Institute for Empirical Aesthetics, Frankfurt, Germany; 2 Center for Language, Music and Emotion, New York University and the Max Plank Institute for Empirical Aesthetics, New York, NY, United States of America; 3 Music and Audio Research Laboratory, New York University, New York, NY, United States of America; Gabriele d’Annunzio University of Chieti and Pescara: Universita degli Studi Gabriele d’Annunzio Chieti Pescara, ITALY

## Abstract

The perception of tension and release dynamics constitutes one of the essential aspects of music listening. However, modeling musical tension to predict perception of listeners has been a challenge to researchers. Seminal work demonstrated that tension is reported consistently by listeners and can be accurately predicted from a discrete set of musical features, combining them into a weighted sum of slopes reflecting their combined dynamics over time. However, previous modeling approaches lack an automatic pipeline for feature extraction that would make them widely accessible to researchers in the field. Here, we present TenseMusic: an open-source automatic predictive tension model that operates with a musical audio as the only input. Using state-of-the-art music information retrieval (MIR) methods, it automatically extracts a set of six features (i.e., loudness, pitch height, tonal tension, roughness, tempo, and onset frequency) to use as predictors for musical tension. The algorithm was optimized using Lasso regression to best predict behavioral tension ratings collected on 38 Western classical musical pieces. Its performance was then tested by assessing the correlation between the predicted tension and unseen continuous behavioral tension ratings yielding large mean correlations between ratings and predictions approximating r = .60 across all pieces. We hope that providing the research community with this well-validated open-source tool for predicting musical tension will motivate further work in music cognition and contribute to elucidate the neural and cognitive correlates of tension dynamics for various musical genres and cultures.

## Introduction

Although musical tension is considered an essential part of the music listening experience, little is known about the underlying mechanisms that trigger its dynamics. Tension increase can be qualitatively defined as the feeling that something meaningful is happening or about to happen in the music and it is closely related to listeners’ expectations in the harmonic or rhythmic structure [1, 2]. When expectations are fulfilled, tension decreases as the music comes to a harmonic resolution [1, 3]. Additionally, tension is commonly associated with arousal, suggesting that tension increases when arousal levels increase [1, 2].

The lack of a unequivocal quantitative definition of musical tension impedes research on its cognitive and neural underpinnings. A quantitative approach could enrich the knowledge about the combination of features contributing to musical tension perception and its inter-subject and intra-subject variability. Ultimately, quantifying musical tension could yield to an improved understanding of the music listening experience, while additionally giving insight into related concepts, such as predictive processing [1, 4] and musical emotions [2]. For example, tension has been suggested to provide the link between low-level musical features and higher level processing such as emotional responses [4]. To advance this central aspect of music cognition, the field would greatly benefit from an automatic prediction model as it allows to computationally characterize how low-level features are critical to emotional arousal. Importantly, most of the available research on musical tension and its correlates has been conducted using western classical music. It is unclear whether tension relies on similar acoustical and musical processes in non-western musical contexts. Quantitatively defining musical tension allows for investigating the mechanisms of tension in western classical music, as well as comparing the experience of tension across musical styles and cultures.

Interestingly, tension ratings are highly correlated across individuals [2, 5], independently of previous experience with the musical pieces [6] or musical preferences [7]. Only minor differences between the tension ratings performed by musicians and those performed by non-musicians have been observed [5]. Critically, even when participants are not provided with an explicit definition of musical tension, they consistently rate musical tension in music, suggesting that they perceive tension as an intuitively accessible aspect of their musical experience [1, 8]. Altogether, converging evidence suggests that tension is an accessible perceptual musical phenomenon modulated by acoustical and musical features [2]. However, the intertwining of such features into the integrative experience of musical tension remains largely under-characterized.

Past research has assessed the impact of a diverse set of features on musical tension using participants’ ratings of tension in excerpts of music. A number of studies have highlighted the association of harmonic and melodic components with tension in a Western classical tonal system [1, 2, 5, 9, 10]. In tonal music, tension is assumed to be at least partly elicited by harmonic dynamics. In this context, models of tonal tension have been developed to capture tension in the harmonic and melodic domain [3]. Previous accounts were able to successfully model tonal tension and show that it is related to behavioral tension ratings [2, 3, 11]. Tonal tension is assumed to consist of several components including sensory dissonance, harmonic instability, and melodic expectation [3]. For example, the tonic in a Western classical context is associated with lower dissonance, high stability, and low melodic expectation, thus it decreases tension. In contrast, highly dissonant intervals or chords are assumed to increase tension [3, 11]. Additionally, notes that strongly lead towards other notes, such as a leading tone to the tonic, seem to increase tension by inducing high levels of instability and melodic expectation [3, 12]. Recent approaches conceptualized tonal tension in the context of the spiral array theory, which quantifies the tonal distances between notes and chords [13]. Here, larger distances between simultaneous notes, successive chords, and chords and the global key induce increased tonal tension [11].

However, the melodic and harmonic characteristics captured by tonal tension only provide one component among several characteristics that make up the experience of musical tension. Besides harmony and melody contour, features such as loudness [1, 2, 10, 14] and tempo [2, 14] have been shown to be consistently related to musical tension. Additionally, onset frequency, which provides a measure of the averaged temporal rate of note onsets at any given time point, was associated with tension in previous investigations [2]. Going beyond the investigation of these feature dynamics in natural music, studies systematically controlling features such as loudness and tempo in selected excerpts revealed interesting insights into the effects of single features and their interactions on perceived tension [1, 2, 14]. Interestingly, while tonal tension seems to rely heavily on musical expectations, other non-tonal features, such as tempo, onset frequency, and loudness modulate tension related arousal [1]. Investigating the relative influence of these two types of features could give insight into the importance of expectations and arousal for tension dynamics. Additionally, tension has been shown to be influenced by timbral features, such as roughness [15, 16]. Roughness is described as the sensation of rapid amplitude fluctuations (so-called beating) resulting from the presentation of two tones with a very small frequency difference [17]. In previous studies, increased roughness has been associated with increased tension [5, 15, 18]. Critically, roughness has been shown to predict tension dynamics in a non-western musical context [16].

Altogether, tension seems to be influenced by combining a set of musical features, which motivated a seminal tension model to integrate loudness, tempo, onset frequency, pitch height, and harmony to successfully predict behavioral ratings of tension [2]. Tension was defined as the combined directional change of these musical features over time. The slope for each feature was computed over an “attentional window” and integrated with a “memory window” capturing the slope of the directly preceding context to account for the build up of tension over long passages of music spanning over several tens of seconds. The prediction model has provided promising prediction and generalization performance in a small selection of pieces stemming from the Western classical domain [2, 19].

This previous model has several shortcomings that are addressed in the current work. The model developed by [2] only works with non-automatically extracted information. To manually perform the feature extraction, users require musical scores or midi files, as well as audio files of the musical pieces. Additionally, the code used for the tension prediction is not available online, which prevents the broad community of interested researchers to use it to their own research questions and data. The set of features used for this original model did not include timbral parameters, despite their proven contribution to overall tension. Finally, the original model has been tested on a very limited set of ten pieces [2], and an updated configuration was only tested on one piece [19]. To provide an ecologically valid prediction model, the model configuration should be based on a larger amount of diverse musical samples.

With this work, we aim to build on the seminal work conducted by [2] while addressing these shortcomings. For the users’ convenience, we provide an open-source python-based tension prediction model that works on the sole input of audio files. The algorithm to predict tension along with example notebooks explaining the procedure are available at https://github.com/vivienbarchet/TenseMusic. Our work includes methods of feature extraction from audio files relying on state-of-the-art music information retrieval (MIR) methods [20]. Furthermore, we provide readers with a repository including the music files used for the model optimization, the feature extraction notebooks, as well as the tension prediction code to enable users to build on our work and adapt the algorithm to their data and research questions. For our model optimization, we use a set of 38 musical pieces stemming from Western classical and modern music to provide a larger corpus of music to inform the model’s optimal configuration. It should be noted that the model presented here is mainly suitable to assess tension in Western classical music. However, it constitutes a valuable tool to investigate tension dynamics in other cultural contexts or musical styles. Investigating model configurations suitable for other musical contexts should be a goal of future research. Notably, future efforts should use non-Western musical stimuli for parameter optimization, in order to evaluate how the model adapts to various musical stimuli. Overall, we hope that our work will motivate further research on the correlates of musical tension in the cognitive, emotional, and the neural domain.

## Model generation

**Fig 1:**
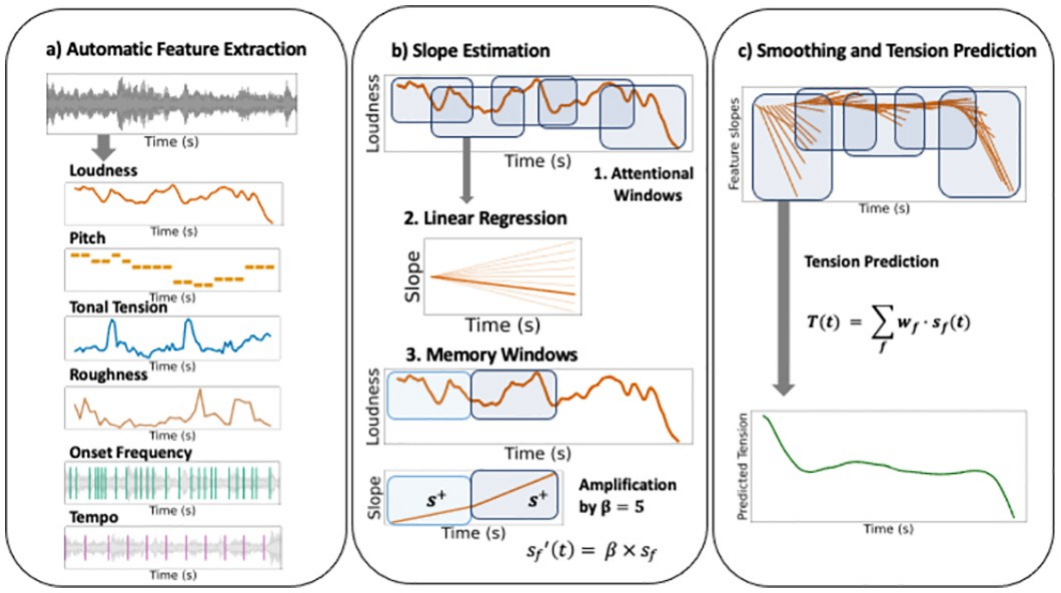
Tension model generation flow. Displayed is a schematic representation of the model including the feature extraction, the tension prediction involving an attentional and a memory window, as well as the global integration of the feature trends. A: The features are extracted automatically using music information retrieval methods in Python. B: To predict tension, the feature time series are divided into sliding attentional windows (Step 1) and the slope of every feature is extracted in each attentional window (Step 2). Each slope is then integrated with the directly preceding slope using memory windows (Step 3). If the direction of the slope in the memory window matches the direction of the slope in the attentional window, the slope is amplified by *β* = 5. C: Tension is predicted from the weighted and summed smoothed feature trends.

We built up on the model architecture from [2] to develop an optimized automatic tension prediction model. It involves a stage of feature extraction from the musical audio files, and a stage of prediction of a tension signal from the combined directional slopes of the features. An overview of the model generation flow is displayed in Fig 1.

The main idea behind this model is that behavioral tension can be predicted from the combined directional change of a set of musical features. The model thus combines the trends of each individual feature at each time point by first estimating each individual feature trend and then integrating the feature trends into a general tension trend. This integration is implemented using sliding attentional windows representing perceptual windows, as well as memory windows that directly precede the attentional windows and are used to integrate the tension trend with the directly preceding context, thus capturing the tension build up over long excerpts of music.

We tested two model versions presented by [2, 19]. The difference between both versions is the method by which the features are integrated into an overall tension trend. In the original model described in [2], the features are assigned individual weights that lead to some features being weighted more heavily than others while all feature trends are captured by the same attentional and memory window size. In an updated model presented by [19], more flexibility is provided by calculating the tension trend in different time scales for each feature, i.e., each feature is assigned an individual attentional and memory window duration.

### Automatic feature extraction

In line with previous work [2], we selected loudness, onset frequency, tempo, pitch height, and harmonic tension as captured by tonal tension as the predictive features. Additionally, we included roughness to investigate the effects of timbral features on tension. We adapted existing MIR tools available in Python, most heavily relying on the Python based library librosa [20]. All features were extracted using Jupyter notebook running on python version 3.9.7.

*Loudness* was extracted using the Zwicker model for temporally variable sounds [21] implemented in the python library MoSQITo [22]. The model provides a perceptually plausible estimate of loudness by taking into account the frequency selectivity of the hearing system, as well as the frequency and the waveform of the sound signal.

*Onset Frequency* was extracted by first identifying the timing of note onsets, using librosa’s note onset detection algorithm. The onset detection is achieved by selecting the amplitude peaks in the envelope. This onset strength envelope is calculated by applying a spectral flux operation on the magnitude spectrogram. The spectral flux describes the increase of spectral energy across the audio by computing differences between consecutive short-term spectra separately for each frequency bin [23]. All positive differences are summed up across frequency bands yielding a one dimensional measure of the amount of increasing spectral energy in the audio [24]. Peaks in the onset strength envelope are then thresholded to reveal the peaks displaying strong increases in spectral energy and most likely corresponding to note onsets. The note durations were inferred by calculating the differences between all successive onset times. To estimate the onset frequency, every event duration was substracted from the maximum event duration in the respective piece. Because the note onset detection is based on the thresholded envelopes’ peaks, any drastic change in the envelopes’ property (such as a change in instrumentation) may result in inaccurate onset detection. Thus, to account for these within-piece inconsistencies and enable a more flexible onset detection, we split the respective audio file in two parts and separately performed onset detection and onset frequency calculation on both parts of the audio.

*Tempo* was estimated using the dynamic tempo estimation method implemented in librosa version 0.9.1 [20]. Dynamic tempo estimation is a challenging task, since multiple hierarchically organized rhythmic levels contribute to an overall tempo perception and a change in the dominant rhythmic structure does not necessarily indicate a change in tempo. Thus, the tempo estimation is based on a cyclic tempogram that identifies equivalent tempi on different rhythm levels that can be shifted in case of a tempo change [25]. The cyclic tempogram was based on a local autocorrelation of the onset envelope used to analyze the local periodicity of onsets and beats.

*Pitch* was extracted using a method for polyphonic pitch estimation. Indeed, all pieces included in the analyses included multiple pitch lines. For polyphonic pitch estimation, we used pretrained models based on neural networks implemented in the python library basic-pitch [26]. For the model input, we calculated the mean of all estimated pitches at each time point in order to receive one aggregated pitch score.

*Tonal tension* was extracted using the model developed by Herremans and Chew [11] based on the spiral array theory [13]. The model is implemented in the python library midi-miner [27]. The midi input required for the estimation of tonal tension was created using the python package basic-pitch [26] that includes a state-of-the-art polyphonic pitch estimation method. According to the model by [11], tonal tension can be quantified using three metrics. For efficiency of computation, we decided to concentrate on the tensile strain, a metric which captures the most reliable and intuitive aspect of tonal tension. The tensile strain captures the tonal distance between the notes contained in a sliding window and the global key estimated for the piece [11].

*Roughness* was used to investigate the contribution of timbral features to musical tension. Roughness is described as the sensation of rapid amplitude fluctuations (so-called beating) resulting from the presentation of two tones whose overtone series include small frequency differences. Roughness has been related to an increased unpleasantness of the sound. In several previous investigations, roughness has been associated with experienced tension [5, 15, 16, 18]. We estimated roughness using the algorithm developed by Daniel and Weber [17] implemented in MoSQITo [22]. All extracted features were z-standardized and smoothed using a moving average filter. The features, originally sampled at 44.1 kHz, were then downsampled to 10 Hz, an appropriate time scale for capturing perceptually relevant phenomena [19]. The code for the feature extraction as well as an example notebook explaining the feature extraction and providing sonification functions to rapidly and intuitively evaluate feature extraction performance can be retrieved from example notebook 1 in https://github.com/vivienbarchet/TenseMusic.

### Slope estimation

The tension prediction is based on the combined directional change of all features, which was captured by feature slopes estimated in overlapping attentional windows, obtained through linear regressions, as depicted in Fig 1b. These slopes were then integrated with the directly preceding context using memory windows leading to a non-linear prediction. This resulted in the slope for each feature at each time point being defined as:

$$\begin{array}{c} s_{f}^{\prime }\left(t\right)=\beta *s_{f}\left(t\right) \end{array}$$ (1)

with *s*_*f*_(*t*) being the slope for every feature *f* at each time point *t*. The *β* parameter enabled the integration of the memory and the attentional windows. If the direction of the tension trend in the attentional window matched the direction of the tension trend in the memory window, *β* was assigned the optimal value of 5 [2]. If the slopes point in different directions, *β* was 1. The slopes in the attentional windows were evaluated using a step size of 250 ms, meaning that a new window began every 250 ms.

### Smoothing and tension prediction

The resulting feature slopes were smoothed using a moving average filter to integrate the overlapping feature slopes at every time point. The smoothed slope for each feature was defined as:

$$\begin{array}{c} S_{f}\left(t\right)=\sum_{\tau =0}^{\frac{d}{h}-1}s\left(t-\tau \cdot h\right)k_{\tau } \end{array}$$ (2)

with *d* being the duration of the attentional window and h being the step size of the trend calculation, which here was 250 ms. *k*_*τ*_ represents a decay constant for a moving average filter, resulting in more recent trends being weighted more heavily. This step was performed at an attentional window size of 3 seconds.

These smoothed feature slopes were then weighted and summed up to receive the final tension prediction. The tension prediction at time *t* was thus defined as

$$\begin{array}{c} S\left(t\right)=\sum_{f}w_{f}S_{f}\left(t\right) \end{array}$$ (3)

with *w*_*f*_ being the feature weights. These weights are optimized using a linear mixed effects model (see section model optimization). Codes for the tension prediction using the optimized weights and window sizes can be retrieved from https://github.com/vivienbarchet/TenseMusic along with an example notebook explaining the tension prediction (example notebook 2).

## Materials and methods

### Participants

Participants were recruited in the New York University psychology undergraduate program and received course credit for their participation. A total of 30 undergraduate students (13 males, age range: 18–34 years (*M* = 20.73, *SD* = 2.87) completed the experiment. All participants reported normal and uncorrected hearing and 17 participants reported that they received formal musical training. These participants reported a wide range of musical experience ranging from 2 years up to 17 years of musical training with a mean of *M* = 7.12 years (*SD* = 4.75). Seven participants indicated that they were still actively practicing music. All participants gave written informed consent prior to starting the study and the procedure was approved by the New York University ethics committee. Six participants were excluded from the analyses since they were unresponsive (i.e., their ratings did not indicate any tension changes in at least two pieces). This exclusion criterion was applied in previous work collecting continuous tension ratings [19].

### Procedure

Following the University policy for restricted in-person experimentation, behavioral data was collected online on the Pavlovia server using Psychopy version 2022.1.4. Participants were instructed to use headphones. Before the start of the data collection, the participants adjusted the volume to a comfortable level while listening to a 20 second excerpt of jazz music. The presentation order of the pieces was randomized across participants. Tension was assessed using continuous ratings, a validated method, used to assess musical tension in numerous previous studies [2, 6, 7, 10, 19]. Prior studies have not included a description of tension as part of the experimental protocol. Indeed, data suggests that listeners have an intuitive comprehension of the concept and how it translates into continuous ratings over the course of a musical piece, as suggested by the remarkable consistency of within and between subjects’ ratings [2, 5]. Here, because the data was collected online and participants had no opportunities to ask any questions during the experiment, we included the following description of rising tension: “the feeling that something important is about to happen in the music”. In doing so, we wanted to avoid confusion between tension and closely related concepts. While listening to the musical pieces, participants were asked to rate their perceived tension on a slider that appeared on the screen and that they controlled with their mouse. The slider ratings were collected at the participants’ monitor frame rate, which corresponded to 60 Hz in the majority of the sample with three participants’ ratings being sampled at higher rates of 120 or 144 Hz. The slider was partitioned into 10 segments of equal size. At the onset of each piece, the initial slider position was fixed at 30 percent of the slider’s width. Additionally, maximum tension was marked at 70 percent of the slider’s width. However, participants could still go beyond the maximum tension level to prevent the tension ratings from being restricted by the slider width. Participants were instructed to use the whole scale for their ratings. Demographic information including age, gender, and musical experience were collected prior to starting the tension rating task. In total, the experiment lasted for approximately 50 minutes.

### Method validation

A mounting number of publications highlighted the value and quality of online data [28]. Several studies have specifically demonstrated that data collected online replicates well established behavioral effects [29–31]. A recent study successfully used online data collection to investigate the effect of musical expertise on various aspects of musical perception, including tension [32]. However, continuous tension ratings, although frequently used in the lab, have not yet been collected online. To validate the methods used in the online data collection and assess the degree of inter-rater agreement, we calculated intraclass correlation estimates across the individual participants for each piece. We calculated an intraclass correlation based on a two-way random effects model to estimate the reliability of the mean of the individual ratings (k = 24). Intraclass correlation estimates and their 95% confidence intervals were obtained using the Python package Pingouin version 0.5.3 [33].

### Stimuli

We collected tension ratings on a diverse set of a total of 38 musical pieces, spanning from baroque to modern atonal music. However, all pieces can be defined as Western classical music and it should be noted that, therefore, our modeled tension remains mainly applicable to Western tonal and atonal classical music. Most of the pieces were orchestral, but the selection also included several solo piano pieces, as well as string quartets and other chamber music ensembles. Some of the pieces were used in a previous study investigating inter-subject correlation during music perception [34]. Participants were presented with a 60 to 90 second excerpt of each piece. Additionally, we included Schubert’s “Morgengruss” –a piece used in a previous study [19]– in the data collection. Participants were presented with the full length piece (approximately 4 minutes), but the piece was cut to 90 seconds for the model training. A complete list of the stimuli can be retrieved from S3 Table. The pieces were sampled at 44,1 kHz when played for behavioral data collection. All pieces were loudness normalized to -23 LUFS using Audacity.

### Data analysis

We calculated correlations between the behavioral tension ratings and the tension predictions to assess the performance of the prediction model. The model was optimized in order to maximize the correlations between the behavioral ratings and the model predictions. To obtain a reliable behavioral tension rating, we first computed the mean of ratings across individual data sets for each musical piece. The z-standardized mean as well as the model predictions were resampled to 2 Hz to calculate correlations, as similar time scales have been used before to assess correlations between time series data [2] and larger rates might lead to overestimated correlation estimates due to the interdependency of the individual time points. Since our time series correlation does not meet the normality requirement of Pearson correlations, we calculated Spearman correlations between the model predictions and the behavioral ratings. Additionally, the correlation analyses incorporated a time shift between the behavioral ratings and the features, since it can be expected that the behavioral ratings lag behind the musical events that trigger tension dynamics. We calculated all correlations with a shift of 4.5 seconds, as it displayed the best correlation performance across pieces.

#### Model optimization

*For the weighted prediction model*, we optimized each feature weight, as well as a global attentional and memory window size. The attentional window duration was varied from 1 to 10 seconds and the memory window duration was varied from 0 to 10 seconds, both in steps of 1 second. The model optimization operated as a two-step process. First, we estimated the optimal combination of attentional and memory windows to predict tension. Thus, we calculated the slopes for every feature captured at each possible combination of attentional and memory window size. These slopes, capturing the features’ dynamics on each time scale, were then entered into a generalized linear mixed model as predictors for the mean tension ratings. The model additionally included a random intercept for the piece. To identify the optimal combination of attentional and memory window size across all features, we used a grouped Lasso regression as implemented in the R library glmmLASSO [35]. The predictors were grouped by time scale in order to reveal one optimal global time scale for all features. The lambda parameter used by the Lasso regression was incrementally adjusted until the model returned one time scale for all features. Secondly, the final feature weights used to predict tension were obtained with a second linear mixed effects model which included only the feature slopes captured on the optimal time window combination resulting from step 1 and a random intercept for the piece.

*For the time scale model*, we again optimized the feature weights but in addition, we optimized the individual attentional and memory window sizes for each feature. Attentional window sizes were varied from 1 to 20 seconds and memory window sizes were varied from 0 to 20 seconds, both in steps of 1 second. Similar to the optimization of the weighted model, we used a two-step process. To estimate the optimal feature window sizes, we first calculated all feature slopes captured on each possible combination of the attentional and memory window size. These feature slopes were entered into a generalized linear mixed effects model as predictors along with a random intercept for the piece. Since, here, we were aiming for individual window sizes for each feature, we did not group the predictors. Instead, to reduce multicollinearity, we calculated an ungrouped Lasso regression. The lambda parameter for this regression was chosen by an iterative procedure in which the parameter was adjusted to obtain the lowest AIC from a wide range of possible parameters. The Lasso regression resulted in a small number of combinations of the attentional and memory window size for every feature displaying non-zero weights. We selected the time window combination with the highest absolute weight for every feature as the optimal individual feature time scale. Secondly, we optimized the feature weights through a linear mixed effects model including the feature slopes captured on the individual time window combinations selected in step 1. The model additionally included a random intercept for the piece.

#### Optimization and validation

To assess the generalization performance of both model variants, we used a leave-one-out cross-validation procedure. Here, we used 38 iterations with each of the iterations including 37 pieces and one piece being left out for the validation step. For each iteration, we performed a model optimization revealing the optimal window sizes and weights for the training data. Then, the predictions generated with these weights and window sizes were correlated with the behavioral ratings for the held-out test piece. In order to provide the optimal model configurations for all pieces, we additionally optimized both model variants on all 38 pieces. An approximate measure of the variance in the tension ratings explained by the feature slopes was obtained using marginal *R*^2^ values for linear mixed effects models [36, 37].

## Results

### Method validation

To assess the degree of inter-rater agreement, we calculated intraclass correlations among the tension ratings for each piece. The analysis indicated high inter-rater reliability for the mean ratings (*M* = .80, *CI*_95%_ = [.78, .82]). This can be classified as good reliability based on commonly used guidelines in the interpretation of intraclass correlations [38]. Hence, this study validates the use of online continuous ratings as a reliable method for research on musical tension on Western musical pieces.

### Model validation

**Fig 2:**
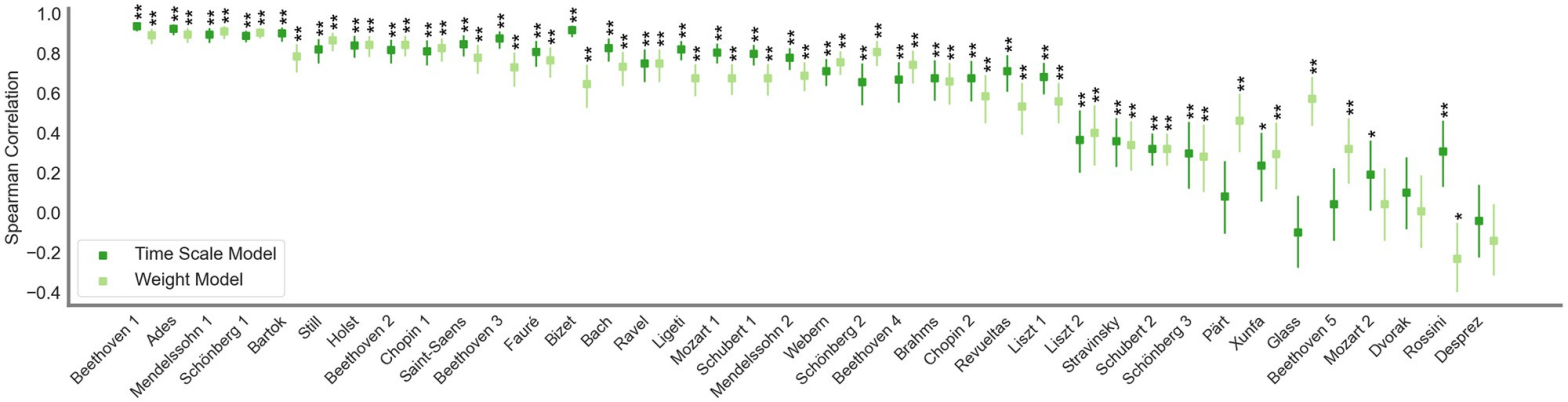
Correlations between the tension ratings and the tension prediction for the test pieces in each cross-validation fold. Displayed are the time-lagged Spearman correlations between the predicted tension and the mean tension ratings. The square dots in dark green indicate the time-lagged Spearman correlation values between the mean tension ratings for the held-out test piece and the tension prediction from the respective validation fold for the time scale model. In light green, the correlations between the tension ratings and the model predictions for the weighted model are plotted. The error bars represent the 95% confidence interval of the Spearman correlations. ** p < .01, * p < .05.

For the weighted prediction model, the cross-validation procedure resulted in a mean correlation between the predicted tension and the averaged tension ratings of *M* = .59 (*SD* = 0.29). The mean RMSE across all iterations was *M* = 0.73 (*SD* = 0.23). For the time scale model, the validation resulted in a mean shifted Spearman correlation of *M* = .61 (*SD* = 0.31) with a mean RMSE of *M* = 0.70 (*SD* = 0.26). The correlations and their confidence intervals are displayed in Fig 2. All correlations for the 38 test pieces, as well as their RMSE can be retrieved from S1 Table. For the time scale model, the correlations for 26 of the 38 pieces (68%) can be classified as large (i.e., *r* > .5) based on traditional conventions in the interpretation of correlations [39]. For another 4 pieces, the correlation estimates can be classified as medium (*r* ≥ .3). For the weighted model, 27 pieces’ correlations (71%) fall into the same range. For another 7 pieces, correlation estimates can be classified as medium. Previous investigations revealed correlations between.60 up to.93 between tension predictions and behavioral ratings [2]. For the majority of pieces, our correlations fall into the same range, further validating the method of data collection. Since we conducted a cross-validation procedure, in which the respective test piece was not included in the model training, our results reveal a convincing generalization performance for the majority of pieces.

However, there are a few pieces for which neither model variant displays significant prediction performance. These are pieces composed by Mozart, Dvorak, and Deprez. Additionally, there are large differences between the performance of both model variants for the pieces composed by Rossini, Glass, Xunfa, and Pärt and one piece by Beethoven. The variable prediction performance might be caused by distinct characteristics of the pieces. These points will be developed in the discussion section.

**Fig 3:**
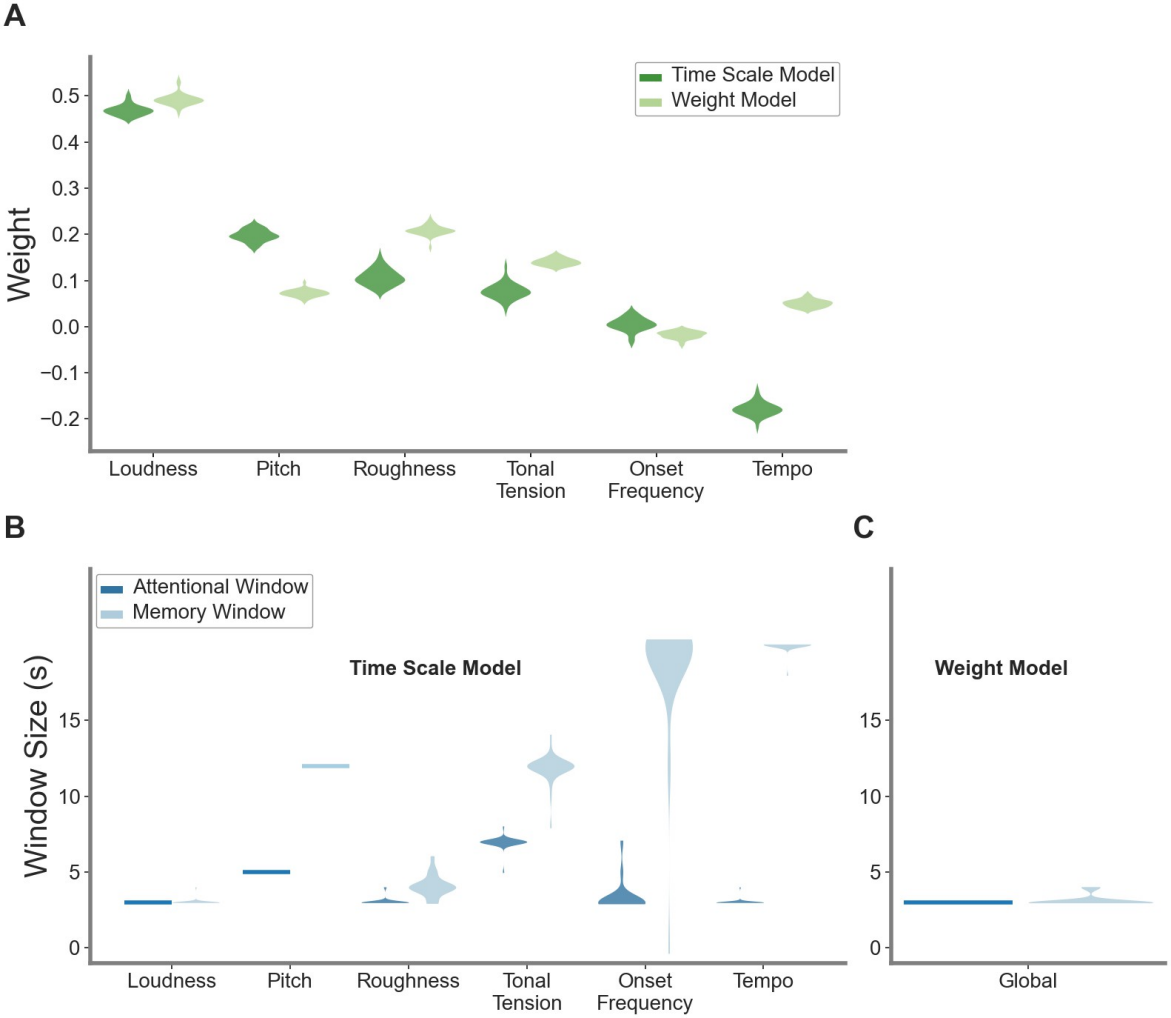
Model generalization. Displayed is the variation of the model parameters, i.e., weights and window sizes, across the 38 cross-validation folds. A: The distribution of the weights for the time scale model is plotted in dark green and the distribution of the weights in the weight model is displayed in bright green. Every dot stands for one cross-validation fold. B: Plotted is the distribution of the feature window sizes for the time scale model. The attentional window sizes are plotted in dark blue and the memory window sizes are plotted in bright blue. C: Plotted is the distribution of the global window sizes for the weighted model. The attentional window sizes are plotted in dark blue and the memory window sizes are plotted in bright blue.

Since the cross-validation procedure yielded 38 slightly different set of model parameters, we assessed their variability across the 38 validation folds. As shown in Fig 3A, the weights overall displayed a low variability across the validation folds. The weights were even more consistent in the weighted model than in the time scale model, and this difference was especially pronounced for the tonal tension, roughness, and tempo features.

The variability of the optimal window duration is shown in Fig 3B. In the weighted model, optimal attentional windows of 3 seconds were assigned in all cross-validation folds. Similarly, the optimal memory window was assigned a duration of 3 to 4 seconds in all folds. This is consistent with previous work applying this model architecture [2]. In the time scale model, loudness was captured by an optimal attentional window of 3 seconds in all foldsand a memory window of 3 seconds in the majority of folds. Similarly, roughness was optimally integrated into the tension trend at attentional windows of 3 seconds and memory windows of 3 seconds in the vast majority of validation folds. Tempo seems to be captured optimally by an attentional window of 3 seconds and a long memory window of around 20 seconds. Onset frequency was optimally captured by attentional windows of around 3 seconds and memory windows of 20 seconds in the majority of validation folds. The optimal window sizes for pitch were consistently set at 5 seconds for the attentional window and at 12 seconds for the memory window in all validation folds. Finally, tonal tension was optimally captured at an attentional window duration of 7 seconds and a memory window duration of 12 seconds in the vast majority of cross-validation folds.

### Final model configuration

**Fig 4:**
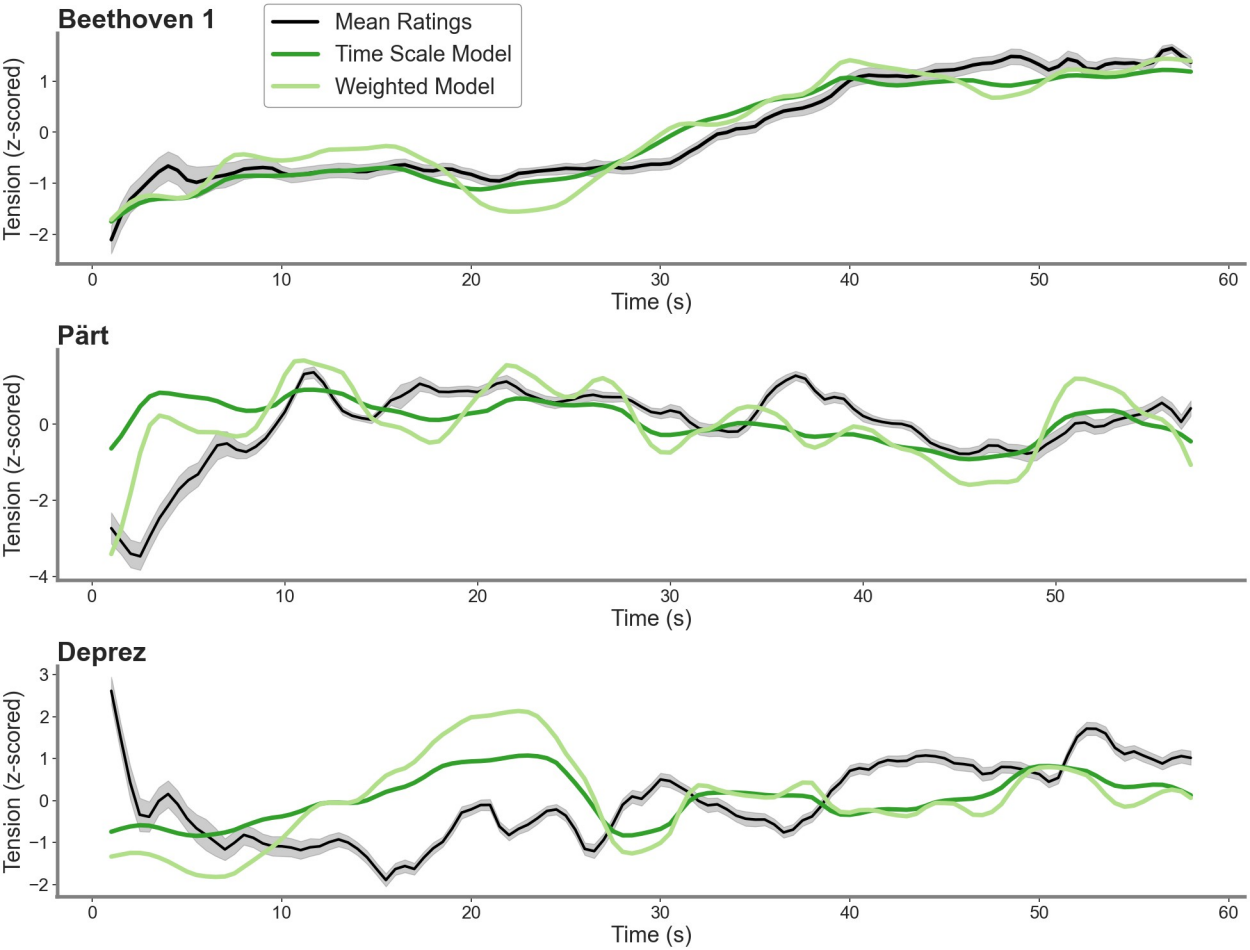
Comparison between the mean tension ratings and the tension predictions from the optimal model configurations. Displayed are the tension predictions and the mean tension ratings for three example pieces taken from our sample. The mean tension ratings are displayed in black. Predictions from the time scale model are plotted in dark green and predictions from the weighted model are plotted in bright green. The error bands show the standard error around the mean of the tension ratings. The mean tension ratings have been shifted by 4.5 seconds to account for the delay in reporting behavioral tension and facilitate the visual evaluation of the overlap between the curves.

The final model was obtained after training the parameters on all 38 pieces, without any cross-validation procedure, in order to include all data available. Predictions from the optimal model configurations for three example pieces with varying correlation with behavior are displayed in Fig 4 alongside with the mean tension ratings. The optimized window sizes and weights are summarized in S2 Table.

#### Weighted model

The optimal window sizes for the weighted prediction model were 3seconds for the attentional window and 3 seconds for the memory window. This is consistent with previous work using the trend salience model [2]. Loudness was assigned the highest weight of all features. Pitch, roughness, and tonal tension received medium positive weights. Tempo was assigned a small positive weight while onset frequency received a small negative weight. The final model prediction displayed a mean correlation of *r*_*spearman*_ = 0.62 (*SD* = 0.27) with the mean tension ratings. The approximate *R*^2^ of the fixed effects was *R*^2^(*marginal*) = 0.46.

#### Time scale model

The optimized weights and window sizes for the time scale model can be retrieved from S2B Table. Loudness, roughness, tempo, and onset frequency were assigned attentional window durations of 3 seconds. Pitch was assigned an attentional window of 5 seconds, whereas tonal tension was optimally integrated using an attentional window of 7 seconds. The optimal memory windows display the most variability across features, with onset frequency and tempo being assigned the longest windows of 20 seconds, respectively. Pitch and tonal tension were assigned medium memory window durations of 12 seconds. Loudness and roughness were assigned short memory windows of 3 and 4 seconds, respectively. The mean correlation of the predictions of the optimized window model and the behavioral tension ratings was *r*_*spearman*_ = 0.68 (*SD* = 0.26). The approximate *R*^2^ of the fixed effects was *R*^2^(*marginal*) = 0.52.

## Discussion

Music is an integral part of most people’s lives, regardless of their cultural origin [40] and therefore, investigating the cognitive and neural processes underlying the human music listening experience is a central topic in human auditory neuroscience [41]. In the Western musical tradition, tension and relaxation dynamics constitute a central part of the music listening experience. In this culture, tension is related to key features of music processing such as expectations and musical emotions [42].

However, the field of music cognition lacks a quantitative description of musical tension due to its multidimensional and phenomenological nature. Indeed, tension seems to be best captured by the listeners’ subjective experience, as continuous ratings, which seems to be driven by certain characteristics of the music [2]. In this work, we present the parameters, algorithmic steps and performance of an automatic tension prediction model that works on the sole input of musical audio files. Our model provides an important tool for future investigations of musical tension addressing the lack of a quantitative description, and an accessible tool to automatically simulate behavioral data. In contrast to existing models [2, 19], we offer an easy-to-use and open-source tension prediction toolbox. We demonstrate that our model displays high prediction and generalization performance for Western classical music. We acknowledge and emphasize that future work should address the model’s generalization performance for other musical styles and cultures. This will also address an existing gap in the literature: the description and function of musical tension in non-Western musical systems [16, 43].

The model combines information from acoustic dynamics, as well as musical features and timbral attributes into a global tension prediction. Loudness provides the highest contribution to the tension prediction across all pieces and both model variants, consistently with previous findings [1, 2]. Additionally, tension is well predicted by roughness, underlining the importance of timbral features for musical tension that has been demonstrated in previous investigations [15, 16]. We additionally demonstrate that pitch height positively predicted tension, which is in line with previous work [2]. Surprisingly, we found that the predictive power of tonal tension was small, given its arguably high relevance for perceived tension in a Western context [3]. However, this echoed more recent investigations, which indicated that tonal tension only provides limited overlap with participant’s tension ratings, which can be attributed to the high contributions of other musical features, such as loudness or roughness [11]. Onset frequency and tempo only contribute to the tension prediction to a limited extent. Tempo was found to be negatively correlated with the tension ratings in some of the pieces (see S2 Fig). This is in line with previous investigations indicating that slower tempo estimates may elicit rising sensations of tension under certain circumstances [2]. However, there does not seem to be a consistent trend in the relation between the tension ratings and the onset frequency.

### Two model variants

We tested the performance of two different model variants. The “weighted model” variant used the same time scale to capture all feature trends together while the “time scale” variant allowed feature integration on flexible time scales. As demonstrated by the large correlations between the tension predictions and the behavioral ratings across cross-validation folds, both models generalize well and accurately predict tension dynamics for unseen pieces.

However, in 8 pieces, neither or only one of the model variants significantly predicted the tension ratings. Four of these pieces are composed in a modern/contemporary (e.g., Glass, Xunfa, Pärt) or early renaissance (e.g. Desprez) musical tradition, and thus may rely on a different combination of features to elicit tension dynamics, if any, which also may yield less consistency in subjects’ ratings. For 4 classical excerpts, we observed low correlations between loudness and the tension ratings, as displayed in S2 Fig. This is very uncommon across studies using tension ratings [1, 2, 10, 14] and results in low correlations with predicted tension. In one of them in particular, by Rossini, the absence of correlations between the tension predictions and the behavioral ratings could be explained by the prominence of percussive instruments, which resulted in an altered extraction of some of the features. Indeed, the broadband envelope for this piece is dominated by non-periodic information, which somewhat hindered the extraction of pitch, dissonance, and onset frequency. These examples emphasize the potential of further model variants that may be tailored to predict tension in pieces from modern composing styles or pieces that do not mainly rely on loudness fluctuations to induce tension. Future work is needed to uncover the different feature combinations that are crucial for eliciting tension in these excerpts.

In spite of this few cases, it should also be emphasized that the model parameters yield high consistency across the validation folds, demonstrating its potential to generalize to other data. From a perceptual viewpoint, the time scale model provides interesting insights into the processing time scales of the different features contributing to tension perception. The most noticeable differences in the window sizes between both model variants are observed for tempo, onset frequency, tonal tension, and pitch. These features display strikingly long optimal memory windows. As a result, the influence of tempo and pitch on the tension prediction is enhanced. This may reflect the higher abstraction level needed to process changes in tempo, tonal tension, and pitch when compared with changes in other features, such as loudness [19]. Perceptible changes in tempo, tonal tension, and pitch could take longer to unfold over time. Tonal tension in particular may rely on the temporal unfolding of harmonic progression over a long time span [19]. The time scale model thus benefits to the flexibility of these varying time of integration, offering a cognitively compelling account of perceived tension.

In conclusion, there is no general trend towards a superior performance of one model variant over the other. However, the time scale model displays slightly higher generalization performance. The generalization performance might benefit from the increased flexibility in the time scale model. The generalization performance however seems to be largely dependent on the characteristics of the pieces tested. To further consolidate the comparison between the two models, future work should address the generalization performance of both variants using training sets of musical samples from different styles, cultures, and genres.

### Limitations

A limitation of this model is that it relies on data collected online. Although previous work used online tension ratings [32], this method is yet not very well established. Our results however are very comparable to previous studies collecting continuous behavioral data in the lab, which suggests that online data collection could be considered a valid technique for future research. In sum, we believe that this model parameters, which have been trained using online continuous tension ratings, will generalize well and predict tension ratings on Western musical pieces collected inside the lab. Higher quality data, collected in an in-person setting, might give additional insights into the combination of individual features. Additionally, the use of physiological measures, such as Electroencephalography (EEG) or Electrodermal activity (EDA) as objective markers of engagement during music listening has recently revealed promising results [34, 44, 45]. Future studies should address the overlap between subjective tension ratings and objective neural data.

Machine learning advances during the last decade have uncovered the potential for computationally complex, non-linear methods (e.g., feed-forward or recurrent neural networks) to explain behavioral and neural data [46, 47]. Both types of neural networks have been extensively tested in the domain of time series prediction [48], which makes them a promising tool for tension prediction. However, their non-linear nature makes them difficult to interpret from a cognitive, perceptually plausible stand-point. The present model operates using state-of-the-art feature extraction and a fairly readable algorithm, which we hope will propel investigations on musical tension and its cognitive and neural basis. Future work using non-linear machine learning techniques to predict tension might provide alternative and important insights on this highly complex perceptual phenomenon.

### Perspectives for future work

We demonstrated the model’s potential to predict tension in music using Western classical composition principles. Future work should investigate tension dynamics in a more diverse set of musical pieces and musical genres. Although previous investigations have shown consistency in ratings for other musical styles [49], so far studies on musical tension dynamics have mainly focused on Western classical music. Few studies have assessed tension in cross-cultural contexts, but they suggest that musical tension depends to some extent on listeners’ musical enculturation [16, 43]. Interestingly, roughness has been shown to predict tension in non-western contexts [16]. Besides, responses to certain features such as loudness have been suggested to be rooted in biological wiring due to its biological functioning as an alarm signal [1]. As such, loudness could be of high relevance to predict tension across musical cultures. These points underline the potential of our approach and feature selection to investigate tension in non-western musical contexts. However, as the experience of tension may be a culture-specific phenomenon, extensive adjustments to the model may be required as data from non-Western music and listeners is collected. This model is perfectly suited to assess the relative influence of the musical features and their relative windows of integration. We hope that providing the research community with an accessible tool for tension prediction will stimulate this research, to tackle the phenomenon of musical tension above and beyond the Western music tradition and explore the interplay of universal and culture-specific feature dynamics in eliciting tension.

In order to provide a sparse and applicable model for tension prediction, the model focuses on a small set of six musical features that have been shown to contribute to the perception of musical tension in prior work [1, 2, 11, 15, 19]. The current work does not exclude the possibility that additional features may also modulate musical tension. Unexplored relevant features may also emerge when considering other musical genres, styles, or cultures. The model provides an informative window into a possible quantitative definition of musical tension in Western classical music, but it is far from being comprehensive or universally applicable. Future work should investigate its suitability and applicability in various musical styles, potentially considering a broader set of features.

## Conclusion

In conclusion, we provide users with a prediction model that accurately predicts behavioral tension ratings from automatically extracted musical features. For the majority of the selected pieces (∼70%), the model displays high generalization performance in a set of Western classical music as indicated by large correlations between the tension predictions and the behavioral ratings. Based on our results, we believe that the model provides a promising tool to investigate tension dynamics in a variety of musical pieces stemming from different genres and styles. However, the low prediction performance in a small set of pieces indicates that additional feature dynamics should be considered, especially for pieces composed in a non-classical Western tradition. We hope that our work will motivate further research into the domain of musical tension to inform a culturally and musically diverse view on musical tension and its cognitive and neural correlates.

## Supporting information

S1 Table Correlations and root-mean squared errors for all cross-validation folds. (PDF)

S2 Table Window sizes and weights for the optimized models. (PDF)

S3 Table Pieces used in the analyses. (PDF)

S1 Fig Comparison between the mean tension ratings and the tension predictions from the cross-validation folds. (PDF)

S2 Fig Correlations between the mean tension ratings and the feature slopes using the window sizes in the optimal model configurations. (PDF)
