# Supplementary material for: TenseMusic: An automatic prediction model for musical tension
Source: PLoS One. 2024 Jan 19;19(1):e0296385. doi: 10.1371/journal.pone.0296385 (PMC10798497; doi:10.1371/journal.pone.0296385)
Supplement: S1 Table — (PDF) [file pone.0296385.s001.pdf]

S1 Table: **Correlations and Root-Mean Squared Errors for all Cross-Validation Folds**.

	**Weighted Model**		**Time Scale Model**	
Test Piece	Correlation	RMSE	Correlation	RMSE
Beethoven 1	0.89**	0.41	0.94**	0.28
Ades	0.9**	0.49	0.93**	0.36
Mendelssohn 1	0.91**	0.53	0.9**	0.37
Schönberg 1	0.91**	0.42	0.89**	0.45
Bartok	0.79**	0.49	0.9**	0.24
Still	0.87**	0.36	0.82**	0.46
Holst	0.84**	0.55	0.84**	0.68
Beethoven 2	0.84**	0.56	0.82**	0.55
Chopin 1	0.83**	0.63	0.81**	0.54
Saint-Saens	0.78**	0.47	0.85**	0.41
Beethoven 3	0.73**	0.59	0.88**	0.43
Fauré	0.77**	0.74	0.81**	0.68
Bizet	0.65**	0.69	0.92**	0.52
Bach	0.73**	0.75	0.83**	0.64
Ravel	0.75**	0.6	0.75**	0.68
Ligeti	0.68**	0.49	0.82**	0.49
Mozart 1	0.68**	0.73	0.81**	0.6
Schubert 1	0.68**	0.71	0.8**	0.62
Mendelssohn 2	0.69**	0.69	0.78**	0.65
Webern	0.76**	0.62	0.71**	0.66
Schönberg 2	0.81**	0.54	0.66**	0.64
Beethoven 4	0.74**	0.7	0.67**	0.7
Brahms	0.66**	0.79	0.68**	0.79
Chopin 2	0.59**	0.69	0.68**	0.62
Revueltas	0.54**	0.83	0.71**	0.7
Liszt 1	0.56**	0.78	0.68**	0.72
Liszt 2	0.4**	0.87	0.37**	0.85
Stravinsky	0.34**	0.97	0.36**	0.95
Schubert 2	0.32**	0.9	0.32**	0.9
Schönberg 3	0.28**	0.6	0.3**	0.56
Pärt	0.47**	0.84	0.08	1.13
Xunfa	0.3**	0.85	0.24*	0.9
Glass	0.57**	0.77	-0.1	1.22
Beethoven 5	0.32**	1.06	0.04	1.21
Mozart 2	0.04	1.14	0.19*	0.99
Dvorak	0.01	1.25	0.1	1.14
Rossini	-0.23*	1.27	0.31**	0.91
Desprez	-0.14	1.25	-0.04	1.19
Mean	0.59*	0.73	0.61	0.7
Standard Deviation	0.29	0.23	0.31	0.26

The table displays Spearman correlations and RMSE values between the model predictions and the behavioral tension ratings of the test piece for each cross-validation fold. These are the correlations between the model predictions and the tension ratings for the pieces that were held out of the optimization procedure.

** $p < .01$

* $p < .05$
