# Supplementary material for: TenseMusic: An automatic prediction model for musical tension
Source: PLoS One. 2024 Jan 19;19(1):e0296385. doi: 10.1371/journal.pone.0296385 (PMC10798497; doi:10.1371/journal.pone.0296385)
Supplement: S2 Table — (PDF) [file pone.0296385.s002.pdf]

S2 Table: **Window Sizes and Weights for the Optimized Models.**

a) Weighted Model

Feature	Weight
Tempo	0.05
Loudness	0.49
Onset Frequency	-0.02
Roughness	0.21
Pitch	0.07
Tonal Tension	0.14

b) Time Scale Model

Feature	Attentional Window (s)	Memory Window (s)	Weight
Tempo	3	20	-0.18
Loudness	3	3	0.47
Onset Frequency	3	20	0.00
Roughness	3	4	0.10
Pitch	5	12	0.20
Tonal Tension	7	12	0.07

The table displays the window sizes and weights for the optimal model configurations. These model configuration resulted from training the model on all 38 pieces in our sample. A: Optimal weighted for the weighted model. B: Optimal windows and weights for the time scale model. In both model variants, loudness was assigned the highest weights. Thus, tension ratings seem to be driven by loudness to a large extent. Interestingly, since longer memory window durations are assigned to tempo and dissonance, their weights seem to increase in the time scale model. Thus, the time scale model allows for a perceptually plausible integration of these features.

** $p < .01$

* $p < .05$
