# Supplementary material for: TenseMusic: An automatic prediction model for musical tension
Source: PLoS One. 2024 Jan 19;19(1):e0296385. doi: 10.1371/journal.pone.0296385 (PMC10798497; doi:10.1371/journal.pone.0296385)
Supplement: S3 Table — (PDF) [file pone.0296385.s003.pdf]

S3 Table: **Pieces used in the Analyses.**

Ades	These Premises are Alarmed
Bach	Cantata, BWV 13: Choral "Der Gott, der mir hat versprochen"
Bartok	Violin Concerto No. 2, 1. Allegro non troppo
Beethoven 1	"Egmont" Overture
Beethoven 2	Symphony No. 4 in B flat major, II. Adagio
Beethoven 3	Symphony No. 2 in D Major, I. Adagio, Allegro con brio
Beethoven 4	Piano Sonata No. 8 in C Minor ('Pathétique'), III. Rondo (Allegro)
Beethoven 5	Symphony No. 5 in C minor, 1. Allegro con brio
Bizet	Symphony No. 1 in C major, 1. Allegro vivo
Brahms	Piano Concerto No. 1, 3. Rondo: Allegro non troppo
Chopin 1	Mazurka No. 13 in A minor
Chopin 2	Nocturne No. 11 in G minor
Desprez	ile fantazies de joskin
Dvořák	Symphony No. 9 in E Minor "From the new world", II. Largo
Fauré	Sonata for Violin and Piano No. 1 in A Major, I. Allegro molto
Glass	String Quartet No. 5 Part 3
Holst	First Suite in E flat Major, III. March
Ligeti	String Quartet No. 1
Liszt 1	A faust Symphony, 3. Mephistopheles
Liszt 2	Danse Macabre
Mendelssohn 1	Symphony No. 1, 4. Allegro con fuoco
Mendelssohn 2	String Quartet No. 3 in D Major, Op. 44 No. 1
Mozart 1	Symphony No. 29 in A major, II. Andante
Mozart 2	Symphony No. 24 in B Flat, K. 182: 1. Allegro Spiritoso
Pärt	Fratres (For Violin and Piano)
Ravel	String Quartet in F Major: II Assez vif
Revueltas	Homenaje a Federico García Lorca
Rossini	La Gazza Ladra, Overture
Saint-Saens	Cello Concerto No. 1 in A Minor, III. Molto allegro
Schönberg 1	5 Orchestral Pieces, Op. 16 No. 5 Das obligate Resitativ
Schönberg 2	Five Orchestral Pieces, I. "Vorgefühle"
Schönberg 3	String Quartet No. 1 in D minor, 3. Mäßig
Schubert 1	Piano Sonata No. 20 in A Major, D. 959: 3. Scherzo (Allegro vivace)
Schubert 2	"Morgengruss" aus Die schöne Müllerin
Still	Symphony No. 1, "Afro-American": 1. Moderato assai
Stravinsky	Piano Sonata (1924), Movement 1
Webern	Symphony, op. 21: II. Variationen
Xunfa	Reaping Crops
