# Supplementary material for: TenseMusic: An automatic prediction model for musical tension
Source: PLoS One. 2024 Jan 19;19(1):e0296385. doi: 10.1371/journal.pone.0296385 (PMC10798497; doi:10.1371/journal.pone.0296385)
Supplement: S1 Fig — (PDF) [file pone.0296385.s004.pdf]

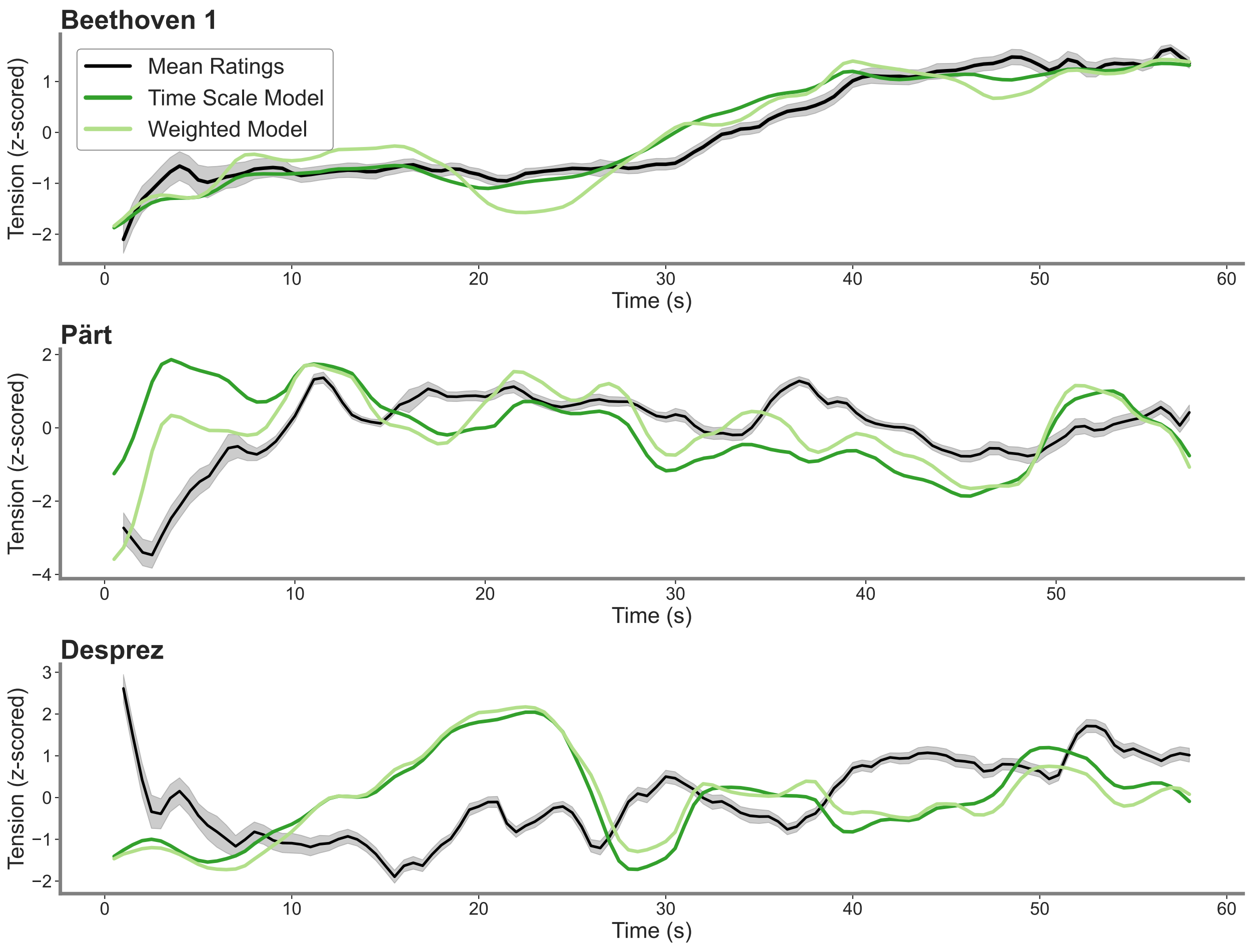

S1 Figure: **Comparison between the Mean Tension Ratings and the Tension Predictions from the Cross-Validation Folds**. Displayed are the tension predictions and the mean tension ratings for three example pieces taken from our sample of musical pieces. The model was trained on all pieces except for the one displayed in the graphs for the respective cross-validation fold. The overlap between the tension ratings and the model predictions underlines the model's potential to generalize to similar pieces. The mean tension ratings have been shifted by 4.5 seconds to account for behavioral delays in behaviorally reporting tension and facilitate the visual evaluation of the overlap between the curves. The error bands show the standard error around the mean of the tension ratings.
