# Supplementary material for: TenseMusic: An automatic prediction model for musical tension
Source: PLoS One. 2024 Jan 19;19(1):e0296385. doi: 10.1371/journal.pone.0296385 (PMC10798497; doi:10.1371/journal.pone.0296385)
Supplement: S2 Fig — (PDF) [file pone.0296385.s005.pdf]

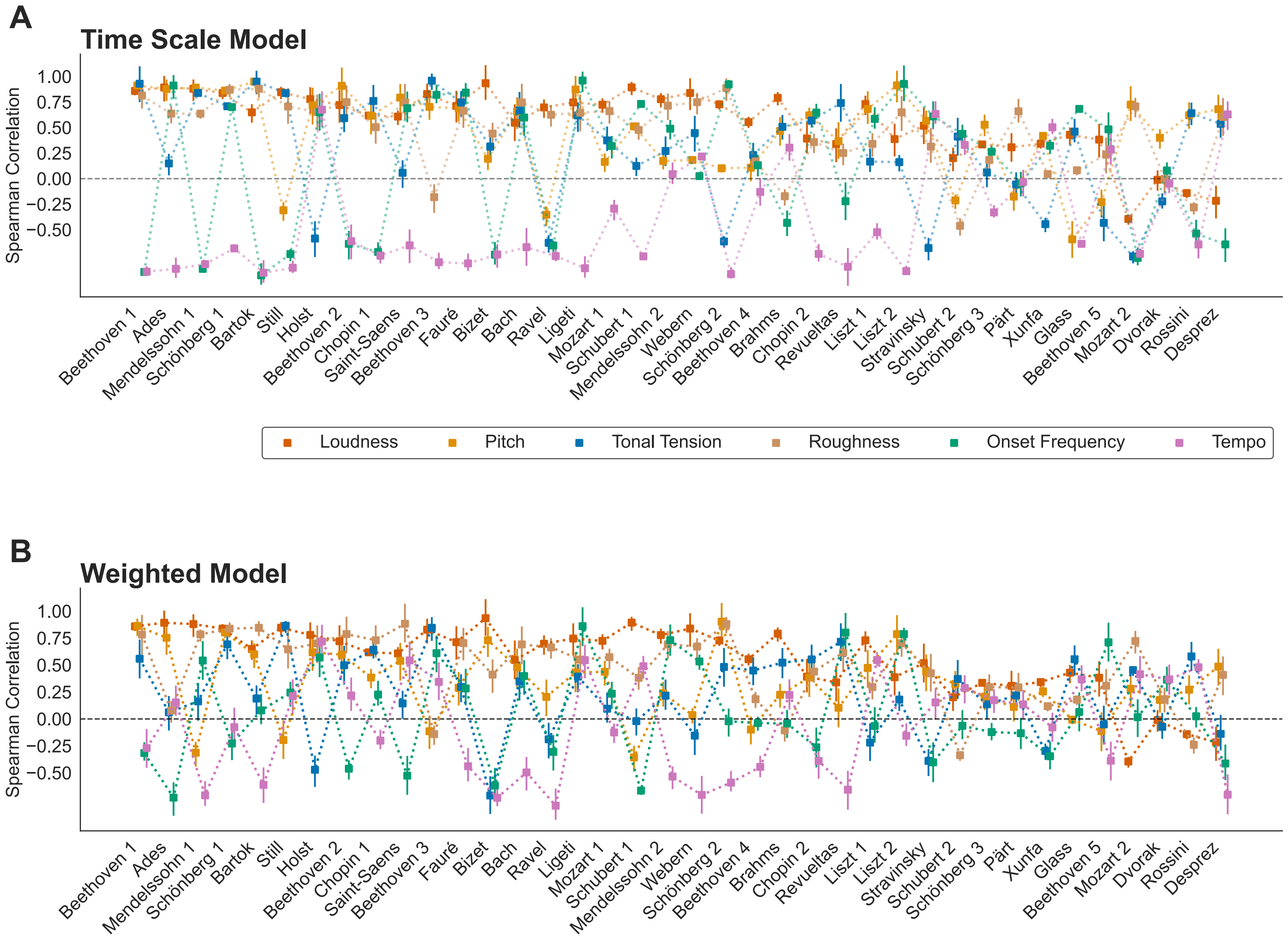

S2 Figure: **Correlations between the Mean Tension Ratings and the Feature Slopes using the Window Sizes in the Optimal Model Configurations**. Displayed are the time-lagged Spearman correlations between the feature slopes and the mean tension ratings. The error bars correspond to the 95% confidence intervals of the correlations. The feature slopes were obtained on the optimal combination of the window sizes revealed for both model variants. A: Correlations between the feature slopes from the time scale model and the mean tension ratings. B: Correlations between the feature slopes from the weighted model and the mean tension ratings. Overall, loudness displays the highest and the most consistent relationship with the tension ratings in both model variants. Onset frequency, pitch, and dissonance display moderate positive correlations with the tension ratings. The correlations between dissonance and tempo and the tension ratings seem to be remarkably more consistent in the time scale model than in the weighted model. By offering more flexibility, the time scale model seems to capture these feature trends significantly better than the weighted model using the same time scales for all feature slopes.
